# Numerical Analysis on Optimal Adhesive Thickness in CFRP Single-Lap Joints Considering Material Properties

**DOI:** 10.3390/ma18112423

**Published:** 2025-05-22

**Authors:** Maruri Takamura, Minori Isozaki, Shin-ichi Takeda, Jun Koyanagi

**Affiliations:** 1 Department of Materials Science and Technology, Tokyo University of Science, 6-3-1 Niijuku, Katsushika-ku 125-8585, Tokyo, Japan; 8217056@alumni.tus.ac.jp (M.T.); 8219012@alumni.tus.ac.jp (M.I.); 2 Aviation Technology Directorate, Japan Aerospace Exploration Agency, 6-13-1 Osawa, Mitaka-shi 181-0015, Tokyo, Japan; takeda.shinichi@jaxa.jp

**Keywords:** CFRP joining, finite element analysis, adhesive thickness optimization

## Abstract

Accurately evaluating the strength of adhesively bonded joints is essential for ensuring structural reliability, but size-dependent effects remain a challenge in consistent strength assessment. This study performs finite element simulations of Single Lap Shear (SLS) tests, focusing on the local stress state at fracture initiation. The analysis considers unidirectional and quasi-isotropic carbon fiber reinforced plastic (CFRP) adherends combined with three adhesives: polyphenylene sulfide (PPS), polyether ether ketone (PEEK), and epoxy. Adhesive thicknesses ranging from 0.1 mm to 0.5 mm are evaluated. The results indicate that the optimal thickness ranges between 0.1–0.3 mm to maximize joint strength, while excessively thin or thick layers reduce performance. These findings align with experimental trends and support the development of precise design guidelines for polymer-based joints in structural applications.

## 1. Introduction

Evaluating the strength of adhesive joints is essential to ensure effective load transfer and to maintain the structural integrity of assemblies. However, current strength evaluation methods for adhesive bonding have limitations, particularly in accounting for the combined stress state at the point of fracture initiation [1]. For example, while the Double Cantilever Beam (DCB) test [2,3] is widely used to assess fracture toughness, the values obtained do not directly correlate with joint strength [4]. Toughness is critical for stable failure modes, but provides limited insight into unstable fractures, where sudden and unpredictable failures occur. On the other hand, the Single Lap Shear (SLS) test offers a more direct approach to assessing joint strength, but the results are highly sensitive to specimen geometry and boundary conditions, which limits its applicability in generalized design criteria [5,6].

To overcome these challenges, this study employs finite element simulations of SLS tests, enabling a more detailed evaluation of joint strength based on stress distributions and fracture initiation behavior. Various joint configurations, including single-lap, double-lap, and step lap joints, have been developed for composite structures, with single-lap joints (SLJs) being particularly popular due to their lightweight design, cost-effectiveness, and ease of fabrication [7,8,9,10,11]. Consequently, SLJs have been extensively studied both experimentally and numerically. Some studies have focused on enhancing joint strength through adhesive modification, such as the incorporation of nanoparticles to improve fatigue resistance and durability [12,13]. Others have investigated the effect of geometrical parameters—particularly adhesive thickness and overlap length—on stress distribution and failure behavior [13,14,15,16,17]. Additionally, several works have conducted finite element simulations or hybrid experimental–numerical analyses to evaluate stress states near the adhesive interface, taking into account factors such as bending effects, damage progression, and failure modes [18,19,20]. These studies collectively demonstrate the versatility and complexity of SLJ configurations, underscoring the need for evaluation methods that reflect the actual stress state at failure initiation.

In our previous research [21], we investigated the discrepancy between experimentally obtained apparent bonding strength and the true bonding strength in the adhesive joints of CFRPs. We demonstrated that, in SLS tests, failure initiation occurs under a combined stress state due to bending effects, resulting in underestimated values for actual interfacial strength. By conducting finite element simulations, we extracted the local stress at the failure initiation point, quantifying the gap between the apparent and true bonding strengths. Additionally, we compared three test methods and found that this approach significantly reduced the discrepancies in strength evaluations while minimizing the influence of adhesive thickness. These findings underscore the importance of considering local stress states and adhesive layer thickness when evaluating joint performance. This study is further motivated by parallel research on ultrasonic welding, where the thermoplastic feature of the energy director plays a crucial role in initiating melting at the interface [22]. While materials such as PPS and PEEK are not typically used as adhesives, they are included in this study because they function as interfacial bonding agents in ultrasonic welding, forming resin layers analogous to adhesive films.

Building on these observations and previous studies, we now review literature on the effects of adhesive thickness. Numerous studies have examined the influence of adhesive thickness on joint performance from both experimental and theoretical perspectives [1,8,23,24,25]. Diharjo et al. [26] studied CFRP-Al joints bonded with epoxy adhesives containing aluminum powder, finding that shear strength peaked at a thickness of 0.4 mm before decreasing. Yang et al. [27] evaluated CFRP joints fabricated with unidirectional prepreg and fabric under room temperature dry (RTD) and elevated temperature wet (ETW) conditions, observing a decrease in strength with increasing adhesive thickness that is more pronounced in ETW environments. Banea et al. [28] investigated polyurethane adhesives and observed a similar trend, though the reduction in strength was less severe than that of brittle adhesives. Grant et al. [29] conducted a comprehensive investigation of SLJs for automotive applications, combining experimental tests with finite element simulations to evaluate adhesive thickness, overlap length, and spew fillet geometry under various loading modes. Their findings highlighted the critical role of shear and bending loads in governing failure modes.

Collectively, these studies confirm that adhesive layer thickness is a crucial factor affecting joint strength across different adhesive types, material systems, and loading conditions. While experimental studies provide valuable insights, they are often constrained by material variability and test-specific limitations. Furthermore, many existing numerical studies employ simplified adhesive models or explore limited parameter ranges, often overlooking the combined effects of adhesive properties, thickness, and failure mechanisms. Recent reviews have also pointed out that microscopic factors, such as stress triaxiality, may influence damage and failure behavior in adhesively bonded composite joints, especially under complex stress states [30].

To address these gaps, the present study investigates the influence of adhesive thickness on joint strength, with particular emphasis on unstable failure behavior and interfacial delamination. In this study, SLJs using CF/epoxy as the adherend material were analyzed. PEEK is a thermoplastic resin that combines mechanical strength with thermal resistance, and it has been increasingly applied in structural bonding of composite materials [31,32]. Finite element simulations of SLS tests were conducted using two types of CFRP adherends, unidirectional and quasi-isotropic, and three types of adhesives: polyphenylene sulfide (PPS), polyether ether ketone (PEEK), and epoxy. Adhesive thickness varied from 0.1 mm to 0.5 mm to determine the optimal configuration for each material system, providing insight into the thickness-dependent failure behavior of CFRP joints.

## 2. Methods

**Figure 1:**
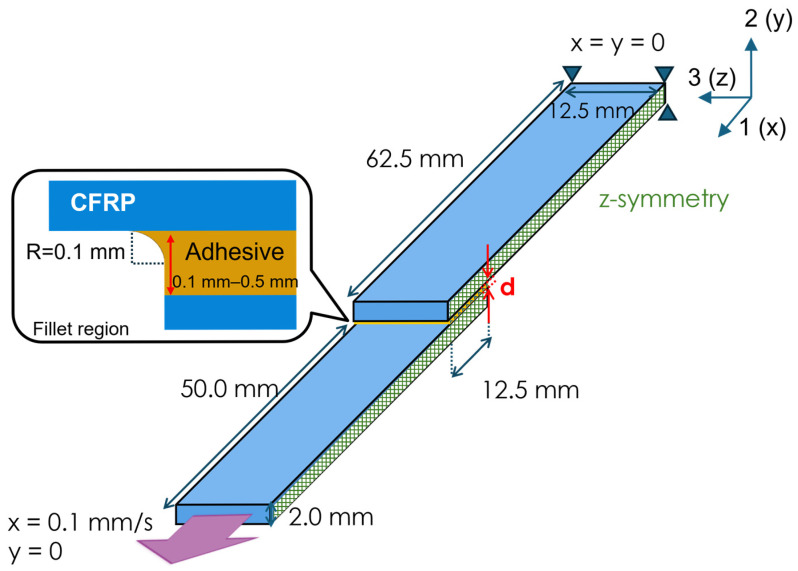
Overview of the finite element model geometry and boundary conditions. Displacement was given in the direction of the arrow. A fillet radius of 0.1 mm was applied at the adhesive edge to reduce stress concentration, and the adhesive thickness was varied from 0.1 mm to 0.5 mm.

Finite element simulations were performed using Abaqus Standard 2020 (Dassault Systemes, Johnston, RI, USA). The model geometry follows ISO standards (ISO 4587:2003) [33]. To reduce computational costs, only half of the structure was modeled in the Z direction. A Z-symmetry boundary condition was applied at the center plane, creating an adhesive area of 12.5 mm × 25 mm for a plate measuring 62.5 mm in length and 12.5 mm in width. A fillet radius of 0.1 mm was introduced at the joint edges (Figure 1) to mitigate stress concentration and reduce mesh dependency.

**Figure 2:**
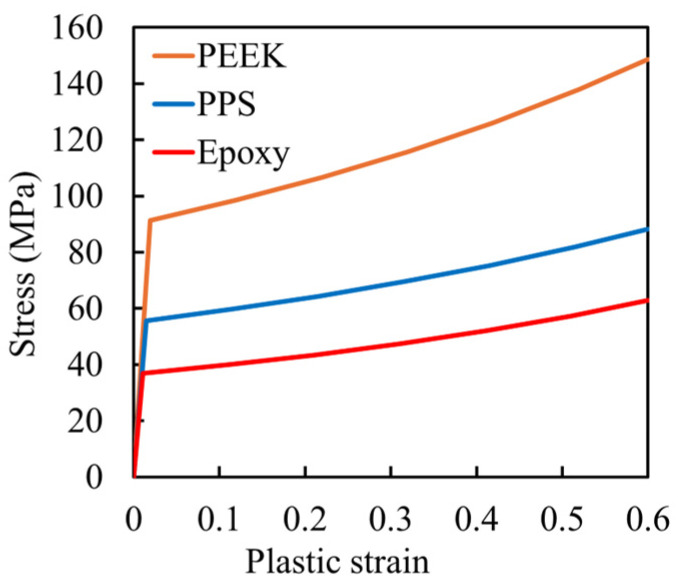
Stress–strain curves representing the plastic behavior of the three adhesive materials (PEEK [35], PPS [36], and epoxy [38]) used in the bonding region of the simulated joints.

Adhesive thickness was treated as a parameter, ranging from 0.1 mm to 0.5 mm in 0.1 mm increments, resulting in five models per material combination. The material properties used in the analysis are detailed in Table 1 and Table 2. Table 1 summarizes the properties of the CFRP adherends, assumed to be CF/epoxy [34]. Two layup configurations were considered: unidirectional (UD) and quasi-isotropic (QI). The UD adherend consisted of [0°]₈ plies, aligned with the loading direction. The QI configuration followed a symmetric layup of [0°/±45°/90°]s. Ply-level properties were homogenized to assign effective orthotropic properties for each layup. The fiber direction of the UD laminate aligns with the displacement direction, corresponding to the 1-axis in Figure 2. Table 2 presents the material properties of the matrix resins (PEEK [35], PPS [36,37], and epoxy [38]) used in the adhesive layer. Plasticity characteristics of each resin are presented in Figure 2. These curves were used to define the damage criteria in the adhesive layer.

Structured hexahedral elements were employed throughout the model. The adherends and adhesive layers were discretized using C3D6T (thermally coupled 6-node) elements. To ensure numerical reliability, a mesh convergence study was conducted. In particular, to accurately capture the stress state near the ends of the adhesive layer where damage initiation typically occurs, local mesh refinement was applied with an element size of 5.0 × 10⁻³ mm.

To monitor interfacial stress distributions without influencing the mechanical response, zero-thickness cohesive elements were inserted at the interface between the adhesive and the adherends. These interface elements were assigned sufficiently high stiffness in all directions and no damage criteria, ensuring they did not affect the stress state of the model and were used solely for post-processing purposes.

**Figure 3:**
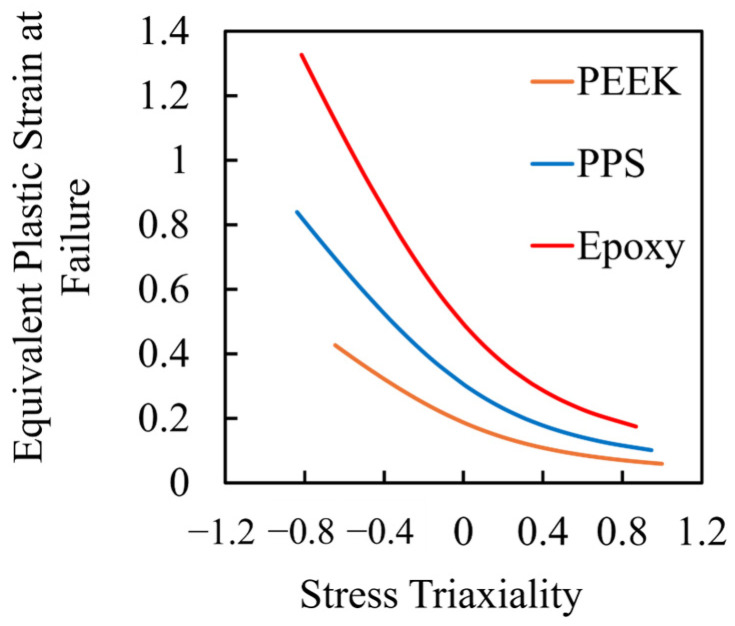
Failure criterion defined by the relationship between equivalent plastic strain and stress triaxiality for each adhesive. Equivalent plastic strain at failure is plotted as a function of stress triaxiality for the three adhesive materials. This relationship serves as the damage initiation criterion used in the finite element simulations.

Failure initiation in the adhesive layer was evaluated using Christensen’s failure criterion [39], which accounts for both the hydrostatic and deviatoric components of the stress state. The failure condition is expressed as:

$$\left(\frac{1}{T}-\frac{1}{C}\right)\left(\sigma_{1}+\sigma_{2}+\sigma_{3}\right)+\frac{1}{TC}\left\{\frac{1}{2}\left[{\left(\sigma_{1}-\sigma_{2}\right)}^{2}+{\left(\sigma_{2}-\sigma_{3}\right)}^{2}+{\left(\sigma_{3}-\sigma_{1}\right)}^{2}\right]\right\}=1$$

where $\sigma_{1},\sigma_{2},\sigma_{3}$ are the principal stresses, T is the tensile strength, and C denotes the tensile and compressive strengths of the adhesive resin, respectively. The first term reflects the contribution of the hydrostatic stress, incorporating the tension–compression asymmetry, while the second term represents a generalized deviatoric stress component based on differences among principal stresses. Failure was assumed to initiate when the left-hand side of the equation reached unity. This formulation enables damage prediction under general multiaxial stress conditions. As illustrated in Figure 3, damage was assumed to occur when the equivalent plastic strain exceeded a critical value corresponding to the given stress triaxiality. Stress triaxiality is defined as the ratio of hydrostatic stress to equivalent von Mises stress, and is often used to characterize the stress state at a point. High triaxiality reflects a stress state that tends to suppress plastic deformation, thereby promoting a brittle material response, even in adhesives that are normally ductile. In contrast, low triaxiality corresponds to a shear-dominated condition that allows for more ductile behavior. The failure condition was applied exclusively to the adhesive layer, cohesive failure was assumed, and the adherends and interfaces were assumed to remain undamaged.

To simulate the residual thermal stress resulting from the difference in thermal expansion between the adhesive and adherends, a two-step analysis was conducted. In the first step, the temperature was reduced from 100 °C to 25 °C, with the assumption that the adhesive and adherends were perfectly bonded. This temperature reduction induced internal thermal stresses due to the mismatch in the coefficients of thermal expansion between the materials. In the second step, mechanical loading was applied by fixing one end of the adherend and displacing the other end until damage initiation occurred in the adhesive layer. The reaction force at the point of damage initiation was used to evaluate and compare the maximum joint strength for each model.

## 3. Results and Discussion

**Figure 4:**
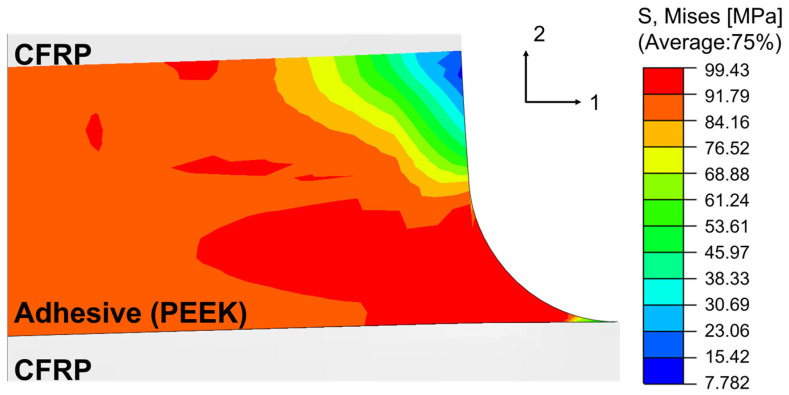
Von Mises stress distribution near the joint edge at the time of predicted damage initiation for a unidirectional CFRP joint bonded with 0.2 mm of PEEK adhesive.

**Figure 5:**
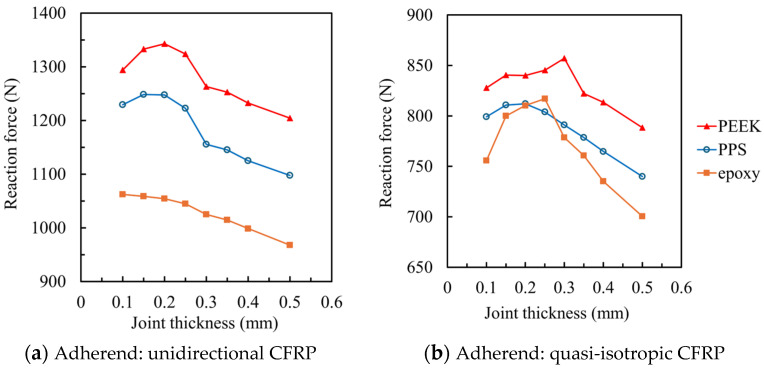
Dependence of adhesive thickness on the reaction force at failure: (**a**) Unidirectional CFRP adherends showing failure characteristics; (**b**) Quasi-isotropic CFRP adherends with comparable behavior.

Damage initiation in all models occurred near the fillet edge. As an example, Figure 4 shows the von Mises stress distribution in the adhesive layer of the unidirectional CFRP joint with 0.1 mm PEEK adhesive at the increment immediately before damage initiation. The stress values represent local peaks rather than average values, and the high stress observed is due to strong stress concentration near the edge of the adhesive layer. This is a typical behavior at failure initiation in bonded joints, and it is consistent with the mechanical strength of thermoplastic adhesives such as PEEK, which was used in other cases in this study. Figure 5 presents the relationship between adhesive thickness and the reaction force at failure for each adhesive–adherend combination. In all cases, an optimal thickness between 0.1 mm and 0.3 mm was identified as yielding the highest joint strength. Thicker bondlines (≥0.5 mm) led to strength degradation due to increased peel stress caused by overlap-end rotation. Conversely, excessively thin bondlines (≤0.1 mm) also resulted in reduced strength, particularly in joints bonded with high-strength adhesives like PEEK and PPS. This phenomenon is attributed to enhanced local constraint at the interface, which can induce higher stress triaxiality, and localized plastic strain accumulation in the adhesive layer. These trends align with experimental observations reported in previous studies [40,41].

**Figure 6:**
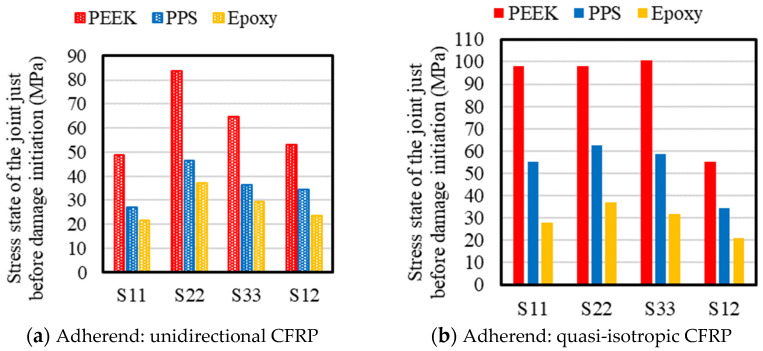
Stress components in the adhesive layer one increment prior to damage initiation (adhesive thickness = 0.2 mm): S11 represents axial stress along the adherend direction, S22 denotes peel stress normal to the adhesive layer, S33 indicates width-direction stress, and S12 corresponds to shear stress.

**Figure 7:**
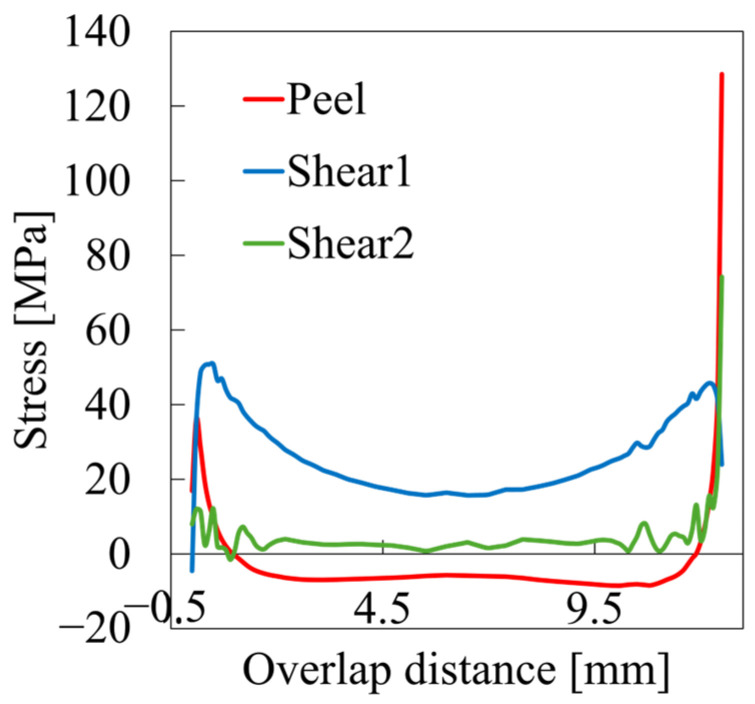
Stress states across the overlap distance in the adhesive layer. Stress concentrations near the adhesive edges are evident, with peel stress being dominant, leading to damage initiation. Shear1 represents shear stress in the direction of applied displacement, while Shear2 indicates the shear stress in the width direction of the specimen. The presented results correspond to the configuration using PEEK adhesive with a thickness of 0.2 mm and unidirectional CFRP adherends. The horizontal axis represents the overlap distance along the x-direction as defined in Figure 1, with the origin (0) corresponding to the end of the overlap where the x-coordinate takes its minimum value.

To better understand the mechanisms leading to failure, we examined the stress distribution immediately before damage initiation for the representative case with 0.2 mm adhesive thickness (Figure 6). Here, S11 represents the tensile stress in the longitudinal direction, S22 represents the stress in the peel direction, S33 represents the stress in the width direction, and S12 represents the shear stress in the test direction. Across all cases, failure was induced by a combination of stress components, with peel stress (S22) being more influential than shear stress (S12) at the failure initiation site. This highlights the influence of bending-induced rotation at the overlap ends, which causes a non-uniform stress distribution and generates significant peel (normal) and tensile stresses in addition to shear, leading to a highly non-uniform stress state. The severity of this peel stress increases with adhesive thickness, as thicker bondlines introduce larger axial offset between adherends. This, in turn, amplifies the bending moment and associated rotation at the overlap ends, resulting in elevated peel stress concentrations. Consequently, the fracture initiation point is subjected not to a pure shear condition, but rather to a complex, multiaxial stress state, particularly in joints with thick adhesives or comparable stiffness between adherends. These findings underscore the inadequacy of estimating joint strength by simply dividing reaction force by the nominal adhesive area and further emphasize the need for numerical simulation to accurately resolve local stress distributions. To validate these insights, the stress state near the adhesive edge was extracted from cohesive elements for the same case (PEEK adhesive, 0.2 mm thickness, UD adherends), as shown in Figure 7. The horizontal axis represents the overlap distance along the x-direction as defined in Figure 1, with the origin (0) corresponding to the end of the overlap where the x-coordinate takes its minimum value. Shear1 represents shear stress in the loading direction while Shear2 denotes shear stress along the specimen width. As supported by prior studies [14,17,42], the high localized stresses observed at the edge are closely tied to failure initiation. These results further highlight the importance of controlling adhesive thickness in practical applications.

**Figure 8:**
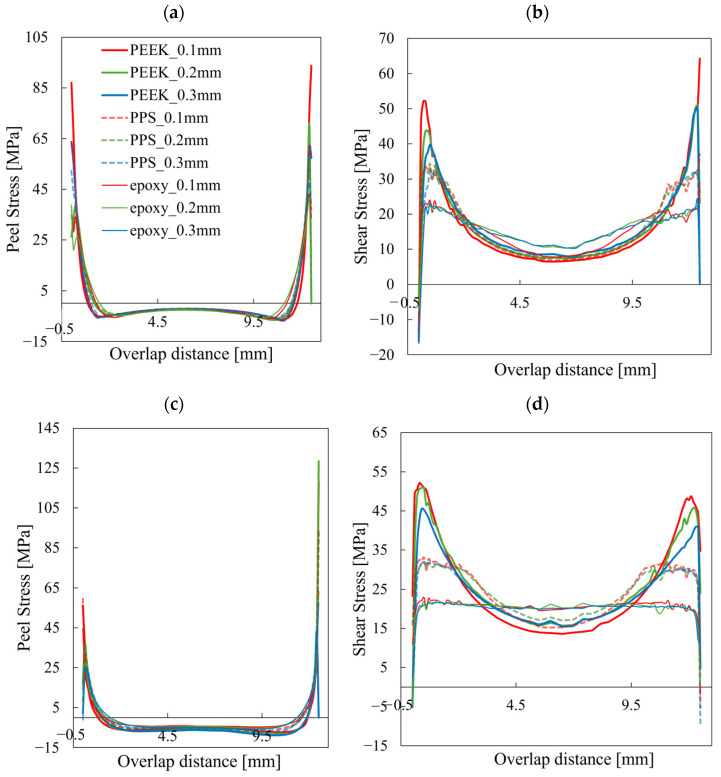
Stress state at the adhesive edge near the onset of damage for various combinations of adhesive type, adhesive thickness, and adherend layup. Peel stress and shear stress in the displacement direction are plotted along the adhesive edge, based on values extracted from cohesive elements one increment prior to damage initiation. The stress profiles reveal how changes in adhesive material, thickness, and adherend layup affect local stress concentrations that govern failure initiation. (**a**) Peel Stress, UD adherends; (**b**) Shear Stress, UD adherends; (**c**) Peel Stress, QI adherends; (**d**) Shear Stress, QI adherends.

Expanding this analysis, Figure 8 illustrates the stress profiles along the adhesive edge for various combinations of adhesive type, adhesive thickness, and adherend layup. The distributions of peel stress and in-plane shear stress (Shear1) reveal several trends. First, peel stress concentrations near the overlap ends are generally more severe than those of shear stress, indicating that peel stress plays a dominant role in damage initiation. This trend is particularly evident in joints with thicker adhesives and quasi-isotropic adherends, where bending-induced rotation is more significant. Second, joints using stiffer adhesives such as PEEK exhibit more pronounced stress localization than those using softer adhesives like PPS or epoxy, suggesting that a higher adhesive modulus intensifies stress concentration at the adhesive edge. Finally, for epoxy-bonded joints, the stress distribution near the edge and the resulting failure behavior appear relatively insensitive to variations in adhesive thickness or adherend layup. In all epoxy cases, failure consistently initiated under a similar stress state, regardless of the joint configuration. These observations demonstrate the importance of considering not only adhesive geometry but also material stiffness and adherend design when evaluating stress localization and joint durability.

**Figure 9:**
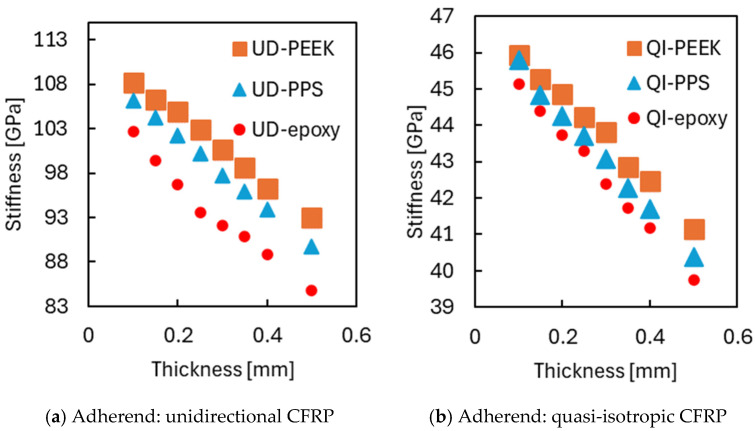
Relationship between overall stiffness and adhesive thickness.

In addition to strength, the joint’s overall mechanical response was evaluated through global stiffness, calculated from the slope of the initial linear portion of the stress–strain curve (Figure 9). Here, stress was defined as the reaction force measured at the end of the joint divided by the cross-sectional area of the adherend at the loaded end, and strain was defined as the overall displacement of the joint divided by its total length. Regardless of adhesive type or layup configuration, stiffness decreased as adhesive thickness increased. This trend reflects the enhanced deformability of the adhesive layer, which becomes more dominant in the joint’s mechanical response as it thickens. Even for stiffer adhesives like PEEK, this effect is evident, indicating that geometric factors can override material stiffness. These observations suggest that while extremely thin layers may raise strength concerns, thinner adhesives are generally recommended for preserving global stiffness.

## 4. Conclusions

This study employed finite element simulations of Single Lap Shear (SLS) tests to investigate the influence of adhesive thickness on the mechanical performance of CFRP joints. By analyzing two types of CFRP layup and three adhesives, an optimal adhesive thickness was identified for each configuration, typically between 0.1 mm and 0.3 mm, beyond which joint strength declined. Stress analysis revealed that damage initiates under a highly non-uniform, multiaxial stress state near the adhesive edge, with peel stress playing a dominant role. This stress concentration intensified with increasing adhesive thickness and in layups prone to bending, highlighting the importance of designing adhesive layer geometry to manage local stress distribution and enhance joint reliability. In terms of global response, joint stiffness decreased with increasing adhesive thickness, regardless of adhesive modulus. This trend underscores a trade-off between stiffness and strength, reinforcing the need to select an appropriate adhesive thickness that balances flexibility and load transfer.

The numerical trends observed were consistent with previous experimental findings, supporting the validity of the simulation approach. These insights offer practical guidance for optimizing bonded composite joints, particularly in weight-critical applications such as aerospace and automotive structures. Future work will involve experimental validation of the simulation results to further confirm the failure mechanisms and optimize joint performance under realistic loading conditions.

## Figures and Tables

**Table 1:** Material properties of CFRP laminates (unidirectional and quasi-isotropic) [34].

	Young’s Modulus [MPa]						Poisson’s Ratio			Density [g/cm^3^]	Specific Heat [J/(kg·K)]	Thermal Conductivity [W/(m·K)]	Thermal Expansion Coefficient Vertical [1/K]	Thermal Expansion Coefficient Shear1 [1/K]	Thermal Expansion Coefficient Shear2 [1/K]
	E1	E2	E3	G12	G13	G23	ν12	ν13	ν23						
Quasi-isotropic	56,800	8210	56,800	3000	4360	3000	0.35	0.25	0.35	1.24	1408	0.72	1.0 × 10^−7^	4.5 × 10^−5^	1.0 × 10^−7^
unidirectional	152,000	8210	8210	4360	4360	3000	0.25	0.25	0.35	1.24	1408	0.72	1.0 × 10^−7^	4.5 × 10^−5^	4.5 × 10^−5^

**Table 2:** Material properties of adhesives.

	Young’s Modulus [MPa]	Poisson’s Ratio	Tensile Strength [MPa]	Compressive Strength [MPa]	Density [t/mm^3^]	Specific Heat [mJ/t·K]	Thermal Conductivity [W/(m·K)]	Thermal Expansion Coefficient [1/K]	Yield Stress [MPa]
PEEK [35]	4600	0.370	100	250	1.30 × 10^−9^	1.00 × 10^9^	0.240	5.00 × 10^−5^	90
PPS [36,37]	3697	0.350	64	160	1.35 × 10^−9^	1.00 × 10^9^	0.290	4.90 × 10^−5^	55
Epoxy [38]	3400	0.396	48	120	1.23 × 10^−9^	1.06 × 10^9^	0.168	6.00 × 10^−5^	36.5

## Data Availability

The original contributions presented in this study are included in the article. Further inquiries can be directed to the corresponding author.
